# The effect of COVID-19 pandemic on admission, management and mortality of pulmonary embolism in cancer patients

**DOI:** 10.1016/j.ijcrp.2026.200580

**Published:** 2026-01-17

**Authors:** Dror Magen, Adam Folman, Marlon V. Gatuz, Rami Abu Fanne, Ariel Roguin, Ofer Kobo

**Affiliations:** a Bruce and Ruth Rappaport Faculty of Medicine, Technion Israel Institute of Technology, Haifa, Israel; b Department of Cardiology, Hillel Yaffe Medical Center, Hadera, Israel; c Keele Cardiovascular Research Group, Keele University, UK

**Keywords:** Pulmonary embolism, Cancer, COVID-19, Pandemic, Outcomes

## Abstract

**Background:**

Pulmonary embolism (PE) is a leading cause of morbidity among cancer patients. The COVID-19 pandemic introduced new challenges to healthcare delivery. This study aimed to evaluate the impact of the COVID-19 pandemic on PE-related hospitalizations, treatment, and in-hospital outcomes in patients with active cancer.

**Methods:**

We conducted a retrospective analysis using the National Inpatient Sample database from 2016 to 2021. Patients with active cancer and a primary diagnosis of acute PE were categorized into three groups: pre-COVID-19 (2016–2019), peak COVID-19 (2020), and ongoing COVID-19 (2021). We compared baseline characteristics, in-hospital procedures, and clinical outcomes among these groups. Multivariable logistic regression was employed to assess associations between COVID-19 periods and outcomes.

**Results:**

Among 170,630 patients with PE and cancer, admission rates decreased during the pandemic. Patients hospitalized during the COVID-19 pandemic more frequently presented with severe PE phenotypes, including saddle PE (9.7 % and 9.4 % vs. 7.5 %, p < 0.001) and acute cor pulmonale (8.4 % and 8.9 % vs. 5.9 %, p < 0.001). Thrombolysis-based therapies increased during the pandemic, whereas adjusted odds of catheter-directed embolectomy were lower. Despite more severe presentations, in-hospital mortality remained relatively stable (6.0 % pre-COVID-19, 6.0 % peak, 5.5 % ongoing; p = 0.004).

**Conclusion:**

The COVID-19 pandemic led to decreased PE-related hospitalizations among cancer patients but was associated with more severe presentations and shifts in therapeutic strategies. Notably, in-hospital mortality remained stable, which may be consistent with maintained PE care pathways during the pandemic. These findings highlight the need for robust, adaptable healthcare systems to ensure continuity of care for high-risk populations during global health crises.

## 1 Introduction

**Fig. 1:**
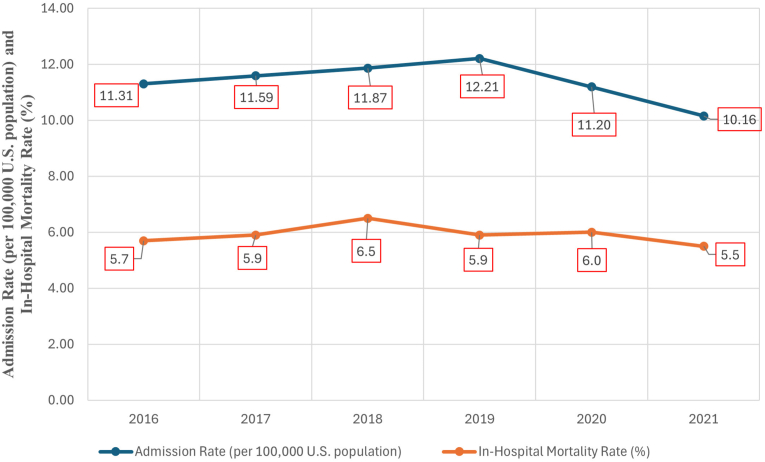
Trends of in-hospital admissions and in-hospital mortality among cancer patients with acute pulmonary embolism from 2016 to 2021.

PE is a life-threatening cardiovascular disorder resulting from thrombotic obstruction of the pulmonary arteries [1]. In cancer patients, PE is a major contributor to morbidity and ranks as the second leading cause of death after the malignancy itself [2,3]. Cancer increases the risk of venous thromboembolism (VTE), including PE, by 4- to 7-fold compared to non-cancer individuals. Approximately 20 % of all VTE cases occur in cancer patients [[4], [5], [6]]. Certain malignancies, particularly pancreatic, lung, and gastric cancers, are associated with higher thrombotic risk [7,8]. Among cancer patients with PE, 30-day all-cause mortality reaches 19.6 %, compared to 3.2 % in non-cancer patients [4] (see Fig. 1).

The COVID-19 pandemic, caused by the SARS-CoV-2 virus, emerged in late 2019 had major social, economic, and healthcare consequences, disrupting societies through lockdowns, isolation, and worsening mental health [9,10]. Hospitals faced overwhelming patient surges, critical shortages of supplies and staff, and widespread postponement of elective procedures [6]. Healthcare systems had to quickly adapt to new infection control protocols, revealing significant vulnerabilities [9].

The emergence of COVID-19 further complicated PE in cancer patients, as the infection itself is associated with a hypercoagulable state, increasing the risk of VTE, including PE [6,8]. Cancer patients, already predisposed to thrombosis, faced heightened vulnerability when infected with SARS-CoV-2 [6]. Beyond the direct thrombotic effects of the virus, the COVID-19 pandemic imposed health systems disruptions that impacted the management and outcomes of PE in cancer patients. Hospital resource strain, delays in cancer diagnosis and treatment, and changes in healthcare delivery all contributed to increased PE-related mortality. During the pandemic, reductions in cancer screening and reporting were observed, potentially leading to later-stage presentations and higher rates of complications such as PE [2]. These findings emphasize that the intersection of cancer, PE, and COVID-19 created a particularly high-risk environment, demanding tailored strategies for thrombosis prevention and management in this vulnerable population [6]. We sought to investigate how the COVID-19 pandemic influenced PE-related hospitalizations, management, and outcomes of PE among cancer patients, utilizing the National Inpatient Sample (2016–2021).

## 2 Methods

### 2.1 Data base

The National Inpatient Sample (NIS), established in 1988, is a publicly accessible database of hospital inpatient stays in the United States, maintained as part of the Healthcare Cost and Utilization Project (HCUP). Since its redesign in 2012, the NIS comprises a 20 % stratified sample of discharges from U.S. community hospitals, excluding rehabilitation and long-term acute care facilities, and captures data from over 7 million inpatient stays annually. It includes all-payer data and is structured to produce nationally representative estimates of hospital utilization, costs, outcomes, and quality of care. This large dataset supports the investigation of rare conditions and enables robust subgroup analyses across geographic and demographic dimensions [11]​. Because this study was conducted using de-identified, publicly available data from the NIS, it was deemed not to constitute human subjects research under U.S. federal regulations (45 CFR 46.102(e 1)). Due to the retrospective nature of the study, Hillel Yaffe Medical Center Institutional Review Board (HYMC IRB) waived the need to obtain informed consent [12].

### 2.2 Study design and population

We conducted a retrospective analysis of adult patients (≥18 years) with active solid or hematologic malignancies hospitalized with a primary diagnosis of PE between 2016 and 2021, categorized into three groups based on time period relative to the COVID-19 pandemic: pre-COVID-19 (2016–2019), Peak COVID-19 (2020), and ongoing COVID-19 (2021). Patient selection was based on the International Classification of Diseases, Tenth Revision, Clinical Modification (ICD-10-CM) diagnosis codes. Table S1 lists the ICD-10 codes related to patient and procedural characteristics. For each hospital discharge, patient demographics such as age, gender, race, admission day (weekday or weekend), expected primary payer, and median household income based on ZIP code were recorded. Records with missing information on age, gender, elective status, admission type and day, and mortality status were excluded from the analysis. First, we assessed the crude PE admission rate per 100,000 US population using data from United States Census Bureau and from the NIS database during the study period, as well as the annual in-hospital mortality rates [13].

### 2.3 Outcomes

The primary outcome was all-cause in-hospital mortality stratified by COVID-19 phases: pre-COVID-19 (2016–2019), Peak COVID-19 (2020), and ongoing COVID-19 (2021). Secondary outcomes, including in-hospital adverse events (major adverse cardiovascular and cerebrovascular events (MACCE), major bleeding, Intracranial Hemorrhage (ICH), non-ICH bleeding events, length of stay and cost) were also evaluated. The MACCE was defined as a composite of all-cause mortality, acute ischemic CVA or transient ischemic attack and cardiac complications. Non-ICH (gastrointestinal, retroperitoneal and procedure-related hemorrhages) were classified as major bleeding events. The treatments that the patients received, including invasive management procedures such as systemic thrombolysis, catheter-directed thrombolysis (CDT), ultrasound-facilitated catheter directed thrombolysis (USCDT), catheter-directed embolectomy (CDE) and surgical embolectomy/thrombectomy, were also examined.

### 2.4 Statistical analysis

Statistical analysis was performed on IBM SPSS version 25. Continuous variables were presented as median and interquartile range, due to skewed data, and categorical data were presented as frequencies and percentages. Pearson's chi-square test was used to compare categorical variables. Sampling weights were used to calculate the estimated total discharges as specified by AHRQ. Multivariable logistic regression models were used to assess the relationship between COVID-19-related time periods and in-hospital clinical outcomes. Odds ratios (ORs) with 95 % confidence intervals (CIs) were produced by these models, adjusted for baseline differences between groups. Adjustment was made for a range of covariates such as age, gender, weekend admission, hospital characteristics cardiogenic shock, heart failure (HF), ventricular fibrillation (VF), ventricular tachycardia (VT), atrial fibrillation (AF), hypertension, hyperlipidemia, diabetes mellitus, valvular heart disease, smoking status, chronic lung disease, chronic liver disease, chronic kidney disease, anemia, thrombocytopenia, coagulopathies, solid malignancies and hematologic malignancies, use of vasopressors, mechanical ventilation, circulatory support (inc. IABP, LV assist device and ECMO) systemic thrombolysis, CDT, USCDT, CDE, surgical embolectomy/thrombectomy.

## 3 Results

During the study period, 170,630 patients with active cancer were hospitalized with a primary diagnosis of acute PE, 116,375 (68.2 %) were hospitalized during pre-COVID-19 period (2016–2019) (11.31–12.21 per 100,000), 28,335 (16.6 %) during peak COVID-19 period (2020) (11.20 per 100,000), and 25,920 (15.2 %) during ongoing COVID-19 period (10.16 per 100,000). The latter has the lowest admission rate since 2016. Table 1 showed the baseline characteristics of the patients during the different periods. Mean age differed slightly across periods with mean age 66.7, 67.1 and 67.9 for pre-COVID-19, peak COVID-19 and ongoing COVID-19, respectively (p < 0.001), as was gender proportion.

**Table 1:** Demographics and baseline characteristics of patients with PE and cancer stratified by COVID-19 time periods.

	Pre-COVID (2016–2019)	Peak COVID (2020)	Ongoing COVID (2021)	P-value
**NIS discharge weight**	116375	28335	25920	<0.001
**Mean Age**	66.7	67.1	67.9	<0.001
**Female, %**	52.1	51.4	52.6	0.017
**Weekend admission, %**	21.0	20.2	20.3	0.003
**Ethnicity, %**				<0.001
White	73.6	74.0	73.6	
Black	16.3	16.5	18.1	
Hispanic	6.1	5.4	4.8	
Asian	1.5	1.5	1.1	
Native	0.3	0.4	0.2	
Other	2.2	2.2	2.3	
**Hospital Region, %**				<0.001
Northeast	20.6	20.9	24.4	
Midwest or North Central	25.6	25.2	28.8	
South	36.5	37.0	42.5	
West	17.2	16.8	4.2	
**Hospital Bed Size, %**				<0.001
Small	18.4	20.1	21.0	
Medium	27.2	26.4	28.0	
Large	54.4	53.5	51.0	
**Hospital Location/Teaching Status, %**				<0.001
Rural	7.7	7.6	7.5	
Urban non-teaching	19.3	16.6	15.5	
Urban teaching	73.0	75.8	77.0	
**Median ZIP income**				<0.001
1st Quartile	26.2	26.1	27.1	
2nd Quartile	25.5	26.9	27.1	
3rd Quartile	26.0	24.9	24.7	
4th Quartile	22.3	22.1	21.1	
**Primary Expected Payer, %**				<0.001
Medicare	58.4	59.0	60.8	
Medicaid	9.8	10.0	8.6	
Private Insurance	27.6	26.9	25.5	
Self-pay	1.7	1.7	2.1	
No charge	0.2	0.1	0.1	
Other	2.2	2.2	2.8	
**Record Characteristics, %**				
Ventricular Fibrillation	0.2	0.2	0.3	<0.001
Ventricular Tachycardia	1.4	1.7	1.6	<0.001
Cardiogenic Shock	1.1	1.5	1.4	<0.001
Saddle PE	7.5	9.7	9.4	<0.001
Acute cor pulmonale	5.9	8.4	8.9	<0.001
Cardiac arrest	1.6	1.9	1.7	<0.001
**Comorbidities, %**				
Heart Failure	12.7	15.3	16.2	<0.001
Valvular Heart Disease	5.0	5.3	5.7	<0.001
Hypertension	60.7	61.7	61.6	0.001
Diabetes Mellitus	23.1	25.2	24.9	<0.001
Hyperlipidemia	35.4	38.9	40.2	<0.001
Atrial Fibrillation/Flutter	13.6	13.7	14.3	0.11
Smoking	44.1	43.9	43.1	0.13
Dementia	3.1	3.6	3.5	<0.001
Chronic Kidney Disease	11.9	14.0	13.9	<0.001
Chronic Lung Disease	26.8	26.0	27.3	0.003
Anemia	39.0	38.8	41.9	<0.001
Thrombocytopenia	10.3	10.1	10.6	0.204
Coagulopathy	7.7	9.7	10.0	<0.001
Chronic Liver Disease	1.2	1.5	1.4	0.001
Peripheral Vascular Disease	3.4	3.2	2.7	<0.001
Previous Acute Myocardial Infarction	4.7	4.8	4.8	0.683
Previous PCI	0.0	0.1	0.1	<0.001
Previous CABG	0.0	0.0	0.0	0.312
Previous CVA	1.0	1.0	1.3	<0.001
Homelessness	0.2	0.4	0.2	<0.001
Hematologic Malignancy	14.6	14.3	14.5	0.457
Solid Malignancy	77.5	78.2	78.0	0.006
Metastatic Malignancy	49.4	49.9	49.2	0.239
Tamponade	0.2	0.3	0.3	0.001

We observed higher rates of saddle PE during peak COVID-19 and ongoing COVID-19 compared to pre COVID-19 (9.7 % and 9.4 % vs 7.5 %, respectively, p < 0.001). Acute cor pulmonale was more frequent between the time periods (8.4 %, 8.9 % vs 5.9 % during peak, ongoing, and pre COVID-19, respectively, p < 0.001). The variations in comorbidities were quite distinct between the different study periods. Heart failure was present in 16.2 % of patients during ongoing COVID-19, 15.3 % during peak COVID-19, and 12.7 % during pre-COVID-19 (p < 0.001). Hyperlipidemia prevalence was increased throughout the time periods with 35.4 %, 38.9 % and 40.2 % during pre-COVID-19, peak COVID-19 and Ongoing COVID-19, respectively (p < 0.001). Coagulopathy prevalence was also increased throughout the time periods with 7.7 %, 9.7 % and 10.0 % during pre-COVID-19, peak COVID-19 and ongoing COVID-19, respectively (p < 0.001). A complete list of comorbidities and their prevalence is provided in Table 1.

## 4 In-hospital procedures and outcomes

### 4.1 Crude rates

Table 2 showed the analysis of in-hospital management and clinical outcomes of patients with a primary diagnosis PE and background cancer comorbidity, stratified by COVID-19 related time periods. There was an increase in CDE use with only 0.8 % during pre-COVID-19, 2.5 % during peak COVID-19 and 4.1 % during ongoing COVID-19 (p < 0.001). Systemic thrombolysis and CDT became less prevalent during ongoing COVID-19 (1.3 and 1.1 respectively) compared to pre-COVID-19 (1.6 and 1.7) and peak COVID-19 (1.7 and 1.8) (p < 0.001).

**Table 2:** In-hospital management and clinical outcomes of patients with Pulmonary Embolism and Cancer Stratified by COVID-19 Time Periods.

	Pre-COVID (2016–2019)	Peak COVID (2020)	Ongoing COVID (2021)	P-value
**NIS discharge weight**	116375	28335	25920	<0.001
**Management, %**				<0.001
Systemic thrombolysis	1.6	1.7	1.3	<0.001
Catheter-directed thrombolysis (CDT)	1.7	1.8	1.1	<0.001
Ultrasound-facilitated catheter-directed thrombolysis (USCDT)	0.4	0.4	>0.1	<0.001
Catheter-directed embolectomy (CDE)	0.8	2.5	4.1	<0.001
Surgical embolectomy/thrombectomy	0.1	0.1	0.1	0.535
**Circulatory and Ventilatory support, %**				
Use of vasopressors	0.9	1.2	1.4	<0.001
Mechanical Ventilation	3.6	3.9	3.4	0.001
ECMO	0.1	0.1	0.1	0.005
**Clinical outcomes, %**				
All-cause mortality	6.0	6.0	5.5	0.004
MACCE	8.9	9.3	9.3	0.007
Major bleeding	4.1	4.4	4.4	0.37
ICH	0.9	1.1	1.0	0.009
Non-ICH, %				
Retroperitoneal	0.2	0.3	0.3	0.067
Gastrointestinal	2.9	2.9	3.1	0.151
Procedure related	0.1	0.1	0.1	0.175
**Length of Stay, days, mean**	5.1	4.9	5.2	<0.001
**Total charge, $, mean**	53554.4	60180.7	62461.8	<0.001

During the study period we observed a slight decrease in mortality during ongoing COVID-19 (5.5 %) compared to pre-COVID-19 and peak COVID-19 (6.0 % for both, p = 0.004). There was a slight increase in MACCE rates during the study period from 8.9 % during pre-COVID-19 to 9.3 % during peak COVID-19 and ongoing COVID-19 (p = 0.007).

Looking into financial aspects, we observed an increase in total charges from $53,554.4 during pre-COVID-19, to $60,180.7 during peak COVID-19 and 62,461.8$ during ongoing COVID-19 (p < 0.001). A complete list of in-hospital procedures and outcomes is provided in Table 2.

### 4.2 Adjusted analysis

The multivariate analysis showed several significant findings (Table 3). The odds of undergoing systemic thrombolysis (aOR 1.306 during peak COVID-19 and 1.300 during ongoing COVID-19, p < 0.001) and CDT (aOR 1.675 during peak COVID-19 and 1.703 during ongoing COVID-19, p < 0.001) were increased. The odds of undergoing USCDT were significantly higher during the peak and ongoing COVID-19 periods. (aOR 10.222 and 11.331) (p < 0.001 for both). In contrast, the odds of going through CDE were decreased during COVID-19 periods (aOR 0.202 during peak COVID-19 and 0.579 during ongoing COVID-19, p < 0.001). The odds of undergoing surgical embolectomy\thrombectomy has increased during peak COVID-19, but has decreased during ongoing COVID-19, both were not statistically significant. In terms of in-hospital complications, peak COVID-19 was associated with higher adjusted odds of in-hospital mortality (aOR 1.192, p < 0.001), whereas ongoing COVID-19 showed no statistically significant difference (aOR 1.085, p = 0.058), despite more severe presentations. MACCE decreased in both peak COVID-19 and ongoing COVID-19, both were not statistically significant. Both Major Bleeding and ICH were relatively stable when comparing peak COVID-19 and ongoing COVID-19 to pre-COVID-19 (not statistically significant). A complete list of multivariate analysis is provided in Table 3.

**Table 3:** Multivariate Analysis showing adjusted OR for in-hospital procedures and complications of patients with Pulmonary Embolism and Cancer Stratified by COVID-19 Time Periods.

**Outcome**	Peak COVID (2020)		Ongoing COVID (2021)	
	**aOR (95 % CI)**	**P value**	**aOR (95 % CI)**	**P value**
**In-Hospital Procedures**				
Systemic Thrombolysis	1.306 (1.155–1.476)	<0.001	1.300 (1.123–1.505)	<0.001
Catheter-Directed Thrombolysis (CDT)	1.675 (1.474–1.903)	<0.001	1.703 (1.468–1.975)	<0.001
Ultrasound-Facilitated Catheter-Directed Thrombolysis (USCDT)	10.222 (5.123–19.151)	<0.001	11.331 (5.945–21.598)	<0.001
Catheter-Directed Embolectomy (CDE)	0.202 (0.185–0.221)	<0.001	0.579 (0.524–0.640)	<0.001
Surgical Embolectomy/Thrombectomy	1.130 (0.681–1.875)	0.637	0.798 (0.428–1.490)	0.48
**Clinical outcomes**				
Mortality	1.192 (1.113–1.276)	<0.001	1.085 (0.997–1.180)	0.058
MACCE	0.964 (0.915–1.014)	0.157	0.968 (0.908–1.032)	0.323
Major Bleeding	1.006 (0.940–1.077)	0.854	1.028 (0.945–1.118)	0.518
ICH	1.094 (0.926–1.292)	0.35	0.936 (0.816–1.075)	0.29

Reference: Pre-COVID (2016–2019); adjusted for age, gender, weekend admission, hospital bed size, region and location/teaching status, cardiogenic shock, heart Failure (HF), ventricular fibrillation (VF), ventricular tachycardia (VT), atrial fibrillation (AF), hypertension, hyperlipidemia, diabetes mellitus, valvular heart disease, smoking status, chronic lung disease, chronic liver disease, chronic kidney disease, anemia, thrombocytopenia, coagulopathies, Solid malignancies and hematologic malignancies, use of vasopressors, mechanical ventilation, circulatory support (inc. IABP, LV assist device and ECMO) systemic thrombolysis, catheter directed thrombolysis (CDT), ultrasound facilitated catheter directed thrombolysis (USCDT), catheter directed embolectomy (CDE), surgical embolectomy/thrombectomy.

## 5 Discussion

We examined the management and outcomes of 170,630 cancer patients hospitalized with PE before COVID-19 pandemic, during the peak of the pandemic (2020) and through the ongoing pandemic period (2021). To our knowledge, this is one of the largest national analysis looking into this specific topic using real national data.

We found that admission rates of PE in cancer patients declined across COVID-19 periods, with 2021 representing the lowest admission rate during 2016–2021. Patients were showing more severe presentations, with Saddle PE and Cor Pulmonale being more frequent. Treatment of PE in cancer patients has shifted, with higher adjusted odds of systemic thrombolysis, CDT and USCDT. While the prevalence of CDE was increased, the odds of undergoing CDE in peak or ongoing COVID-19 have decreased. Despite more severe PE presentations in peak and ongoing COVID-19, in-hospital mortality was similar across periods. Secondary clinical outcomes, such as MACCE, major bleeding or ICH, had either decreased or stayed relatively stable.

Because NIS captures inpatient admission, we cannot determine whether lower PE-related admission reflects a true reduction in PE incidence or reduced admission without a corresponding change in incidence. Lower admission may relate to decreased healthcare seeking during the pandemic, pandemic-related restrictions limiting access to hospitalization and/or higher thresholds for inpatient admission. Patients who were ultimately admitted may have represented a more selected, clinically severe PE phenotype, making them more likely to seek medical attention and/or to pass the hospitalization threshold during the pandemic, whereas patients with milder PE may have delayed care or preferred to remain at home. A study by Pelletier et al. showed a decrease in overall hospitalizations in the US when comparing Peak COVID-19 to pre-COVID-19 [14]. This overall hospitalization trend might also be relevant to PE hospitalization, suggesting the decrease of PE hospitalization might be connected to an overall hospitalization decrease, and not specifically to a PE incidence decrease. Regarding PE hospitalization, Gottlieb et al. reported a similar trend as in our study in which PE admission rate in the general population decreased between 2019 and 2021, with the admission rate in ongoing COVID-19 being the lowest in the period of 2016–2021, that is consistent with our study [15]. Bansal et al. showed that the incidence of PE hospitalization in the general population in Cleveland hospitals between pre-COVID-19 to peak and ongoing COVID-19 has increased, in contrast to this study (p = 0.478 for pre-COVID-19, p < 0.001 for peak and ongoing COVID-19) [16]. Çinier et al. reported fewer STEMI cases undergoing primary PCI during lockdown and longer pain-to-balloon and door-to-balloon time, implying delayed or absent presentation of high-risk patients [17]. Similarly, Çınar et al. showed that frail, comorbid COVID-19 patients with low prognostic nutritional index had substantially higher in-hospital mortality [18], supporting the possibility that our study represents cancer-PE patients who successfully accessed inpatient services, while some of the most vulnerable patients, such as ones with concurrent COVID-related myocarditis, acute myocardial infarction, or embolic complications, may be underrepresented.

A more severe PE presentation among cancer patients during the pandemic may reflect two possibilities: either severity truly increased during the COVID-19 period, or hospitalizations preferentially captured higher-acuity cases while the underlying severity distribution in the population remained unchanged. This possible shift in the hospitalized case-mix may reflect pandemic-era changes in healthcare access and/or changes in admission thresholds, preferentially capturing higher-acuity presentations. Tilliridou et al. demonstrated that cor pulmonale trends during the COVID-19 pandemic were inconsistent. In April, the incidence increased from 47.6 % in the pre-COVID period to 69.2 % during the COVID peak (p = 0.376), whereas in May it decreased from 60.0 % to 58.6 % (p = 0.029) when comparing the same periods [19]. Finn et al. examined PE presentation in the general population at New York-Presbyterian Hospital/Columbia University and observed an increase in saddle PE cases during peak COVID-19 compared to pre-COVID-19 (17.4 % vs. 20.9 %, p = 1), a finding consistent with our study. However, unlike our findings, the incidence of cor pulmonale in the specified cohort remained stable across the two periods (65.1 % vs. 65.2 %, p = 0.99) [20].

In our study, a notable shift in therapeutic strategies was observed during the COVID-19 period, characterized by a significant increase in the utilization of systemic thrombolysis and CDT, accompanied by lower adjusted odds of CDE. This transition may reflect adaptations to pandemic-related constraints, including guidelines aimed at minimizing patient transport and invasive procedures to reduce exposure risks, thereby reserving interventions for only the most critical cases. Thrombolysis-based therapies, which can be administered more rapidly and require fewer resources, are particularly advantageous in settings with limited personnel and equipment. Their lower associated costs and comparable in-hospital length of stay, further justify their preferential use during periods of staffing shortages and restricted access to operative suites, especially amid a high prevalence of high-risk PE cases [1,[21], [22], [23], [24], [25]]. Consistent with these observations, Finn et al. reported an increase in systemic thrombolysis use (OR 3.9, p = 0.3) and a decrease in the use of CDE (OR 0.16, p = 0.16), in alignment with our findings. In contrast to our study which demonstrated an increase in CDT usage, CDT remained relatively stable in this specified study (OR 1.06, p = 1)^20^. In a study conducted by Shalaby et al., which evaluated clinical outcomes in PE among cancer patients compared to non-cancer patients between 2002 and 2014, it was found that cancer patients had lower odds of receiving systemic thrombolysis (OR 0.68, p < 0.0001). Conversely, cancer patients exhibited higher odds of vasopressor administration compared to their non-cancer counterparts (OR 1.25, p < 0.0001), indicating a potentially more severe hemodynamic profile in this population [26].

According to our findings, despite the substantial health system strain imposed by the COVID-19 pandemic, including hospital resource limitations, staffing shortages, and modifications to healthcare delivery, the all-cause in-hospital mortality among cancer patients admitted with PE remained relatively stable. Notably, this stability persisted even as patients increasingly presented with more severe PE phenotypes, such as saddle PE and acute cor pulmonale, indicating a possible adaptation of PE care pathways in order to maintain stable in-hospital mortality among cancer patients. One plausible explanation is the prioritization of life-threatening conditions, such as PE, by hospitals, which likely maintained essential diagnostic and therapeutic protocols despite pandemic-related constraints [20,21]. Moreover, while elective and routine care experienced widespread delays during this period, emergent conditions like PE likely continued to receive urgent and guideline-directed management, facilitated by standardized care pathways and reinforced by national treatment protocols [[20], [21], [22]]. In contrast to our findings, Bansal A et al. reported an increase in PE-related mortality within the general population in the Cleveland hospitals during the covid pandemic, when compared to mortality in acute pe patients before the COVID-19 pandemic [16]. However, the combination of lower crude admissions and higher adjusted mortality may reflect a shift in the hospitalized case mix toward more severe presentations during the pandemic, such that in-hospital mortality may not fully reflect risk across the broader cancer-PE population. Finn et al. observed an increase in the use of vasopressors during the pandemic period, corresponding to our findings, with Finn et al. reporting an odds ratio of 4.9 (p = 0.006). We observed a relatively stable prevalence in the use of ECMO and mechanical ventilation, whereas Finn et al. reported increased utilization of these interventions, with odds ratios of 5.02 (p = 0.33) and 4.2 (p = 0.04), respectively [20]. Our findings also underscore the importance of strengthening PE prevention efforts in high-risk populations. Hu et al. suggested that non-communicable diseases (NCDs), mainly cardiovascular disease, DM and cancer frequently coexist and may be interrelated, highlighting the importance of Cardiovascular-DM-Cancer (CDC) strips for early primary or secondary prevention. Hu et al. further proposed expansion to the “Re-CDC strips” framework incorporating respiratory disease [27]. This multimorbidity pattern is consistently observed in our study across pre-COVID-19, peak COVID-19, and ongoing COVID-19 periods, including DM (23.1 %, 25.2 % and 24.9 % respectively, p < 0.001) and respiratory comorbidity (26.8 %, 26.0 % and 27.3 % respectively, p = 0.003). Collectively, these findings support the clinical significance emphasized by Hu et al. regarding the significance of CDC strips as well as the Re-CDC expansion in prevention strategies and risk-factor control in high-risk populations.

Overall, our findings emphasize the need for robust thrombotic risk management and sustained access to timely PE diagnosis and treatment for high-risk cancer patients during healthcare disruptions such as the COVID-19 pandemic, supporting consideration of durable strategies to maintain continuity of thrombosis care in vulnerable populations, including safeguarding essential diagnostic and therapeutic interventions to mitigate adverse outcomes.

## 6 Limitations

Despite the highlighted clinical application, this study has several limitations. First, the study is a retrospective observational design using administrative data from the National Inpatient Sample (NIS), which is subject to coding inaccuracies and potential biases inherent in secondary data analysis. Second, the reliance on administrative coding (ICD-10-CM) introduces the potential for misclassification and coding inaccuracies, which may affect the validity of case identification and outcomes. Additionally, the dataset lacks critical clinical details such as laboratory values, PE severity score imaging findings, cancer stage, and anticoagulation status, which constrain the ability to perform detailed risk stratification or understand underlying mechanisms. The study also did not conduct a dedicated sub-analysis of cancer patients with concurrent PE and COVID-19, thereby limiting insight into the specific impact of SARS-CoV-2 infection on this high-risk subgroup. Although we adjusted for multiple covariates, residual confounding from unmeasured clinical factors cannot be excluded. Additionally, because NIS data lacks lifestyle and dietary variables, we were unable to evaluate prevention-focused approaches. Hu C emphasized that a “healthy E(e)SEEDi lifestyle” improves life quality and human immunity and may help prevent or suppress NCDs, including cardiovascular disease and cancer [28]. In addition, Hu C discusses marine natural products (MNPs) as “huge, novel, and promising” biomedical resources, describing their potential relevance to SARS-CoV-2–related cardiovascular disease and possible mechanisms linked to human immunity [29]. As these exposures and preventive strategies are not captured in NIS, their potential role in PE prevention among cancer patients could not be assessed in the present study. Despite these limitations, the study's strengths include a comprehensive analysis of a large, nationally representative NIS dataset, enabling evaluation of a diverse patient population across multiple healthcare settings and thereby enhancing the generalizability of findings to real-world inpatient practice.

## 7 Conclusion

In conclusion, our study of a very large nationwide population suggested that the COVID-19 pandemic was associated with changes in PE-related hospitalizations and management among cancer patients. Although PE admissions declined during the pandemic years, patients admitted during this period exhibited more severe clinical presentations and a greater reliance on advanced interventional therapies. Despite these challenges, in-hospital mortality rates remained stable, which could be consistent with maintained, potentially adaptive PE care pathways, despite healthcare disruptions. Our findings reinforce the importance of maintaining robust PE pathways even amid healthcare crises. Future efforts should focus on sustaining access to timely diagnostic and therapeutic services and developing targeted thromboprophylaxis strategies to mitigate the compounded risks posed by concurrent cancer and COVID-19.

## CRediT authorship contribution statement

**Dror Magen:** Writing – review & editing, Writing – original draft, Visualization, Investigation, Formal analysis. **Adam Folman:** Writing – review & editing, Visualization, Validation. **Marlon V. Gatuz:** Software, Resources, Data curation. **Rami Abu Fanne:** Validation. **Ariel Roguin:** Validation. **Ofer Kobo:** Validation, Supervision, Project administration, Methodology, Conceptualization.

## Declaration

Ethics approval and consent to participate: This study used de-identified patient data from the publicly available National Inpatient Sample (NIS) database, maintained by the Healthcare Cost and Utilization Project (HCUP). In accordance with the U.S. Department of Health and Human Services policy for the protection of human research participants, the use of this dataset does not constitute human subject research. The study was reviewed and approved by the Hillel Yaffe Medical Center Institutional Review Board (HYMC IRB). The IRB waived the need for obtaining informed consent due to the retrospective nature of the study. All methods were carried out in accordance with relevant guidelines and regulations.

## Consent for publication

Not applicable.

## Availability of data and materials

The data that support the findings of this study are from the Healthcare Cost and Utilization Project (HCUP) National Inpatient Sample (NIS) database. This dataset is available for purchase from HCUP at https://www.hcup-us.ahrq.gov/nisoverview.jsp and https://cdors.ahrq.gov/databases. The dataset used in this analysis was obtained under a data use agreement and cannot be publicly posted. Access to the raw data requires completion of a data use agreement and purchase from HCUP. De-identified data used in this study can be made available upon reasonable request to the corresponding author, Dr. Ofer Kobo (ofermkobo@gmail.com), subject to HCUP's data use policies.

## Funding

The authors did not receive support from any organization for the submitted work.

## Declaration of competing interest

The authors declare that they have no competing financial interests or personal relationships that could have appeared to influence the work reported in this paper.
