# Supplementary material for: The effect of COVID-19 pandemic on admission, management and mortality of pulmonary embolism in cancer patients
Source: Int J Cardiol Cardiovasc Risk Prev. 2026 Jan 17;28:200580. doi: 10.1016/j.ijcrp.2026.200580 (PMC12861057; doi:10.1016/j.ijcrp.2026.200580)
Supplement: Multimedia component 1 [file mmc1.docx]

**Table S1:** ICD-10 codes for patient characteristics, in-hospital procedures and post-procedural complications

| Category | Description | | | ICD-10 Codes |
| --- | --- | --- | --- | --- |
| Primary Diagnosis | Acute Pulmonary Embolism (PE) | | | I26 |
|  | Saddle PE with Cor Pulmonale | | | I26.02 |
|  | Saddle PE without Cor Pulmonale | | | I26.92 |
|  | Acute Cor Pulmonale | | | I26.01, I26.02, I26.09 |
| Study Groups | Active Cancer (Solid Malignancy) | | | C00 - C80, C7A, C7B |
|  | Active Cancer (Hematologic) | | | C81 - C96 |
| Record Characteristics  and  Comorbidities | Ventricular Fibrillation | | | I49.1 |
|  | Ventricular Tachycardia | | | I47.2 |
|  | Cardiac arrest | | | I46 |
|  | Cardiogenic Shock | | | R57.0 |
|  | Hypertension | | | I10 - I15 |
|  | Valvular disease | | | I05-08, I34-3 |
|  | Diabetes Mellitus | | | E08 - E13 |
|  | Heart Failure | | | I50 |
|  | Atrial Fibrillation | | | I48 |
|  | Chronic Kidney Disease (CKD) | | | N18 |
|  | Chronic Lung Disease (COPD) | | | J44 |
|  | Coagulopathy | | | D65 - D68, D69.0-69.2 |
|  | Dementia | | | F01 - F03 |
|  | Chronic Liver Disease | | | K73-K74 |
|  | Homelessness | | | Z59 |
|  | Tamponade | | | I31.4 |
|  | Hyperlipidemia \ Dyslipidemia | | | E78 |
|  | Anemia | | | D50 - D59 |
|  | Thrombocytopenia | | | D69.2 - D69.6 |
|  | Obesity | | | E66 |
|  | Smoking | | | Z87.891, Z72.0 |
|  | Peripheral Vascular Disease | | | I73.9 |
| Procedures (PCS)  and  Outcomes | Systemic Thrombolysis | | | 3E03317 |
|  | Catheter-Directed Thrombolysis (CDT) | | | 3E06317 |
|  | Ultrasound-Facilitated CDT | | | 6A750Z5, 6A750Z6, 6A750Z7, 6A750ZZ, 6A751Z5, 6A751Z6, 6A751Z7, 6A751ZZ |
|  | Catheter-Directed Embolectomy (CDE) | | | 02CP3ZZ, 02CQ3ZZ, 02CR3ZZ |
|  | Surgical Embolectomy | | | 02CP0ZZ, 02CQ0ZZ, 02CR0ZZ |
|  | Use of vasopressors | | | 3E030XZ, 3E033XZ, 3E040XZ, 3E043XZ |
|  | Mechanical Ventilation | | | 5A19054, 5A1935Z, 5A1945Z, 5A1955Z |
|  | Circulatory support (inc. IABP, LV assist device and ECMO). | | | 5A02x, 5A1522G, 5A15A2G, 5A15A2H |
|  | MACCE (Composite) | All-cause mortality | | Not coded as an ICD-10 outcome (captured via in-hospital death status) |
|  |  | Acute ischemic CVA | | I63 |
|  |  | Cardiac complications | Coronary artery dissection | I25.42 |
|  |  |  | Pericardial effusion (incl tamponade) | I23.0 I31.2 I31.4 I31.3 |
|  |  |  | Tamponade | I31.4 |
|  |  |  | Dressler‘s syndrome | I24.1 |
|  |  |  | Post MI angina | I23.7 |
|  |  |  | Intracardiac Thrombus | I23.6 |
|  |  |  | Mechanical complications | I23.1-I23.5 |
|  | Major Bleeding | | | R58, D62, K92.2 |
|  | ICH | | | I60 - I62 |
|  | Non-ICH | Retroperitoneal | | K66.1 |
|  |  | Gastrointestinal | | K92.0 - K92.2, K25.0 - K25.2, K25.4 - K25.6, K26.0 - K26.2, K27.0 - K27.2, K27.4 - K27.6, K28.0 - K28.2, K28.4 - K28.6 |
